# Generation of human chronic wasting disease in transgenic mice

**DOI:** 10.1186/s40478-021-01262-y

**Published:** 2021-09-26

**Authors:** Zerui Wang, Kefeng Qin, Manuel V. Camacho, Ignazio Cali, Jue Yuan, Pingping Shen, Justin Greenlee, Qingzhong Kong, James A. Mastrianni, Wen-Quan Zou

**Affiliations:** 1 Department of Pathology, Case Western Reserve University School of Medicine, Cleveland, OH 44106 USA; 2 Department of Neurology and Center for Comprehensive Care and Research On Memory Disorders, The University of Chicago Pritzker School of Medicine, Chicago, IL USA; 3 Virus and Prion Research Unit, Agricultural Research Service, National Animal Disease Center, USDA, 1920 Dayton Avenue, Ames, IA 50010 USA; 4 Department of Neurology, Case Western Reserve University School of Medicine, Cleveland, OH 44106 USA; 5 National Prion Disease Pathology Surveillance Center, Case Western Reserve University School of Medicine, Cleveland, OH 44106 USA; 6 National Center for Regenerative Medicine, Case Western Reserve University School of Medicine, Cleveland, OH 44106 USA

**Keywords:** Chronic wasting disease (CWD), Prion disease, Prions (PrP^Sc^), Cellular prion protein (PrP^C^), Serial protein misfolding cyclic amplification (sPMCA)

## Abstract

Chronic wasting disease (CWD) is a cervid prion disease caused by the accumulation of an infectious misfolded conformer (PrP^Sc^) of cellular prion protein (PrP^C^). It has been spreading rapidly in North America and also found in Asia and Europe. Although bovine spongiform encephalopathy (i.e. mad cow disease) is the only animal prion disease known to be zoonotic, the transmissibility of CWD to humans remains uncertain. Here we report the generation of the first CWD-derived infectious human PrP^Sc^ by elk CWD PrP^Sc^-seeded conversion of PrP^C^ in normal human brain homogenates using in vitro protein misfolding cyclic amplification (PMCA). Western blotting with human PrP selective antibody confirmed that the PMCA-generated protease-resistant PrP^Sc^ was derived from the human PrP^C^ substrate. Two lines of humanized transgenic mice expressing human PrP with either Val or Met at the polymorphic codon 129 developed clinical prion disease following intracerebral inoculation with the PMCA-generated CWD-derived human PrP^Sc^. Diseased mice exhibited distinct PrP^Sc^ patterns and neuropathological changes in the brain. Our study, using PMCA and animal bioassays, provides the first evidence that CWD PrP^Sc^ can cross the species barrier to convert human PrP^C^ into infectious PrP^Sc^ that can produce bona fide prion disease when inoculated into humanized transgenic mice.

**Supplementary Information:**

The online version contains supplementary material available at 10.1186/s40478-021-01262-y.

## Introduction

Prion diseases are fatal transmissible spongiform encephalopathies of humans and animals characterized by the accumulation of the infectious prion protein (PrP^Sc^) that is derived from its cellular isoform (PrP^C^) through a structural transition [32]. It is known that prion diseases are less transmissible across species because of species barrier associated largely with differences in each host’s PrP sequences [31]. However, bovine spongiform encephalopathy (BSE, commonly named mad cow disease) has been well-documented to cause variant Creutzfeldt-Jakob disease (vCJD) in humans [8, 13, 41], the first proven zoonotic prion disease. The outbreak of mad cow disease and its zoonotic transmissibility raise concerns about the potential public health threat from other animal-derived prion diseases.

Chronic wasting disease (CWD) is the most contagious of all prion diseases, and it is endemic in North America, having spread to 26 US states and 3 Canadian provinces. The disease has also been found in South Korea and most recently in Europe [6, 38]. The CWD prevalence rates among free-ranging cervids are as high as 40% in some areas of Colorado, Wyoming, and Wisconsin of the United States, where large amounts of venison are consumed [21, 30, 36]. Moreover, prions are readily shed from infected cervids through urine, feces, saliva, and carcasses. Prions in these excreta and bodies remain stable and infectious in the environment for many years. Thus, CWD poses potential risks to public health in North America. However, the zoonotic potential of CWD remains uncertain. On the one hand, there has been no published epidemiological evidence to support CWD transmissibility to humans. Published transmission studies so far have consistently failed to transmit the CWD agent to “humanized” transgenic (Tg) mice expressing human PrP [20, 23, 37]. Moreover, Race and co-workers have reported that the non-human primate Cynomolgus macaques were not susceptible to CWD [33–35]. On the other hand, Barria and co-workers observed that human PrP^C^ from normal humanized transgenic (Tg) mouse brains could be converted by CWD PrP^Sc^ into proteinase K (PK)-resistant PrP (PrP^res^), albeit at low to moderate conversion efficiencies [3, 4]. In addition, squirrel monkeys have been found to be highly susceptible to CWD from white-tailed deer, mule deer, and elk after either intracerebral or oral challenges [3, 4, 27]. Furthermore, a recent international study observed an atypical phenotype in Cynomolgus macaques after challenge with CWD prions orally or intracerebrally: the affected animals showed minimal PrP^Sc^ in the CNS by immunohistochemistry staining and positive prion seeding activity by RT-QuIC and PMCA assays [14]. These conflicting experimental data and observations preclude a clear answer on whether CWD prions are zoonotic at this point. In order to demonstrate that CWD prions are zoonotic, it will be critical to search for direct evidence that CWD prions are capable of converting human brain PrP^C^ into infectious PrP^Sc^, that CWD prions can infect Tg mice expressing human PrP, and that ultimately the first cases of CWD transmissions to humans are identified. In addition, generation of experimental human CWD prions will also provide critical clues for the detection and diagnosis of acquired human CWD cases if and when they occur.

It is worth noting that the annual number of sporadic CJD (sCJD) cases in the USA has increased, with the total number of suspected and confirmed sCJD cases rising from 284 in 2003 to 511 in 2017 (https://www.cdc.gov/prions/cjd/occurrence-transmission.html). The greatly enhanced CJD surveillance and an aging population in the USA certainly contributed to the observed increase in annual sCJD case numbers in recent years, but the possibility cannot be excluded that some of the increased sCJD prevalence is linked to CWD exposure.

In the present study, using serial protein misfolding cyclic amplification (sPMCA) assay we generate PrP^Sc^ by seeding CWD prions in normal human brain homogenates. Importantly, we reveal that two lines of humanized Tg mice expressing human PrP-129VV and 129MM develop prion diseases upon intracerebral inoculation of the abnormal PrP generated by sPMCA. We believe that our study provides the first opportunity to dissect the clinical, pathological and biochemical features of the CWD-derived human prion disease in two lines of humanized Tg mice expressing two major human PrP genotypes, respectively.

## Materials and methods

### Reagents and antibodies

Proteinase K (PK) was purchased from Sigma Chemical Co. (St. Louis, MO). Reagents for enhanced chemiluminescence (ECL Plus) were from GE Healthcare. Anti-PrP antibody 3F4 against human PrP residues 107–112 [18, 45] and horseradish peroxidase-conjugated sheep anti-mouse IgG were purchased from Millipore Sigma (Burlington, MA).

### Preparation of brain samples

Frozen non-CJD human brain tissues with PrP^C^-129MM or PrP^C^-129VV were collected at autopsy and maintained at -80 °C until used as substrates in PMCA. Brain tissues from uninfected humanized TgMM or TgVV, cervidized TgDeer (kindly provided by Glenn Telling, Ph.D., Colorado State University), or hamsters were also used as substrates in serial PMCA as controls. The samples were rinsed with PBS three times to avoid blood contamination before homogenization. For sequencing of the functional cervid PrP genes, genomic DNA was purified from frozen cervid brain tissues using standard phenol–chloroform extraction, and the coding region from the PrP genes was amplified by PCR using DePrP223 (ACACCCTC-TTTATTTTGCAG) and DePrP224 (AGAAGATAATGAAAACAGGAAG) primers. The PCR products were cloned into pstBlue-1. Purified PCR product and multiple clones from each cervid DNA sample were subjected to sequencing by MCLAB (South San Francisco, CA, USA) using the same primers. The elk samples came from elk with different *PRNP* genotypes: #1, #2, #3, and #4 (CWD-negative) were PrP-132MM; #5, #6 and #7 were PrP-132ML; and #8 was PrP-132LL. The genotype of the deer CWD (dCWD) isolate #9 was not available. Elk samples #1 through #7 and deer sample #9 were from captive animals in the terminal stages of disease and originated from the western United States. Elk sample #8 was from an elk with the PrP-132LL genotype that was experimentally inoculated with brain homogenate from a farmed elk of the same genotype.

Brain tissues were homogenized with a mini-Beads Beater in conversion buffer containing 150 mM NaCl, 1% Triton X-100, 8 mM EDTA and a complete protease inhibitor in PBS without Ca^2+^ and Mg^2+^ for a 10% brain homogenate. The samples were then centrifuged at 500 g for 5 min. The supernatant (S1) was transferred to a clean tube for future use. Brain homogenates (10%, weight/volume) from elk (n = 7, #1-#3, #5-#8) and deer (n = 1, #9) with CWD or non-CWD (n = 1, #4) were prepared as PMCA seeds in 1 × lysis buffer containing [10 mM Tris–HCl, 150 mM NaCl, 0.5% Nonidet P-40 (NP-40), 0.5% deoxycholate, 5 mM ethylenediaminetetraacetic acid (EDTA), pH 7.4]. For prion-infected mouse brain tissues, 10% brain homogenates were prepared in 1 × lysis buffer and the brain homogenates were treated with designated amounts of PK prior to Western blotting probing with 3F4. To prepare the detergent-soluble (S2) and detergent-insoluble (P2) fractions, the PMCA products or 10% normal brain homogenate (substrate, in conversion buffer) were mixed with an equal volume of 2 × lysis buffer and then subjected to ultracentrifugation at 100,000 g for 1 h at 4 °C. The supernatant was transferred into a clean tube as the S2, whereas the pellet was resuspended in 1 × lysis buffer as the P2 at the volume equal to that of S2.

### Protein misfolding cyclic amplification (PMCA)

The preparation of PrP seeds and substrates, as well as PMCA, were conducted as previously described [11, 40] and above. Each seed was diluted in the substrate at a ratio of 1:100 (1 µL seeds + 99 µL substrates containing 50 µg/mL heparin) into a 200 µL PCR tube with 1 PTFE beads (diameter 3/32’’) (Teflon, APT, RI). 20 µL of each mixture was taken and kept at − 20 °C as a non-PMCA control. The remaining mixture was subjected to serial PMCA (sPMCA). Each cycle was comprised of a 20 s elapse time of sonication at amplitude 85 (250 watts; Misonix S3000 sonicator) followed by an incubation period of 29 min 40 s at 37 °C. Each round of sPMCA consisted of 96 cycles. For subsequent sPMCA rounds, 10 µL sample was taken from the last cycle of the immediate preceding round and placed into 90 µL fresh normal brain substrates to start a new round of amplifications.

### Prion-inoculation and monitoring of humanized transgenic mice

Two lines of humanized transgenic mice were used in this study for assessing the infectivity of the PMCA-induced CWD-derived human PrP^Sc^. Humanized TgMM (also named Tg40h) and TgVV (also termed Tg1307, derived from Tg152) mice expressing wild-type human PrP carrying either 129 M or 129 V polymorphism without endogenous mouse PrP as previously described [20, 42] were used in this study. The PMCA product seeded by #6 CWD prion isolate was diluted into 1% human brain homogenate in 1 × PBS before inoculation. After anesthetization of Tg mice with isoflurane, 30 µL of the diluted PMCA product was injected intracerebrally into each mouse with a 26-gauge needle as described previously [20, 42]. After intracerebral inoculations, the animals were monitored at least 3 times per week for symptoms such as coarse coat, waddling gait, hunched back, tail plasticity, and bradykinesia. Within 2–3 d days after presentation of clear symptoms or at death, the brain was removed and one-half brain was frozen for Western blotting of PrP^Sc^, and the other half was fixed in formalin for histology and immunohistochemistry analysis as described below.

### Western blotting

PMCA brain samples were subjected to treatment with PK at 100 µg/mL for 70 min at 45 °C with agitation prior to Western blotting. Samples were resolved on 15% Tris–HCl Criterion pre-cast gels (Bio-Rad) for SDS-PAGE as described previously [44]. The proteins on the gels were transferred to Immobilon-P membrane polyvinylidene fluoride membrane (PVDF, Millipore) for 2 h at 350 mA. For probing of PrP, the membranes were incubated for 2 h at room temperature with anti-PrP antibody 3F4 at 1:40,000 as the primary antibody. Following incubation with horseradish peroxidase-conjugated sheep anti-mouse IgG at 1:5,000, the PrP bands were visualized on Kodak film (Millipore Sigma) by ECL Plus as described by the manufacturer (GE Healthcare). For detection of the PK-resistant PrP^Sc^ in the frozen brain tissues of infected Tg mice, 50 µg/mL of PK or designated amounts of PK were used as indicated in each experiment at 37 °C for 1 h.

### Histology, immunohistochemistry and lesion profiles

Histological and immunohistochemical evaluations were carried out on sagittal brain levels at approximately 3.12 mm and 1.56 mm to the midline as previously described [9, 40]. Paraffin-embedded sections were stained with hematoxylin–eosin (H&E) or immunohistochemistry (IHC) probed with the 3F4 antibody at 1:1,500 dilution, 10% GVM and then processed with the DAB detection kit as described by the manufacturer. Lesion profiles were generated following semi-quantitative evaluation of spongiform degeneration (SD) severity, which was rated on a 0 to 3 scale on H&E-stained Sects. (0: not detectable; 1: mild, 2: moderate, and 3: severe). The eight brain regions examined included the cerebral cortex (CC), hippocampus (HI), basal ganglia (BG), thalamus (TH), dorsal (d) and ventral (v) midbrain (MB), inferior brainstem (BS.i), and cerebellum (CE).

### Statistical analysis

The differences in incubation times between TgVV and TgMM mice inoculated with sPMCA-induced CWD-derived human PrP^Sc^ as well as differences in lesion severity between different mice were statistically analyzed using Student’s *T*-test to obtain *p* values for comparisons between two groups.

## Results

### Generation of CWD-derived human PrP^Sc^ by PMCA

**Fig. 1:**
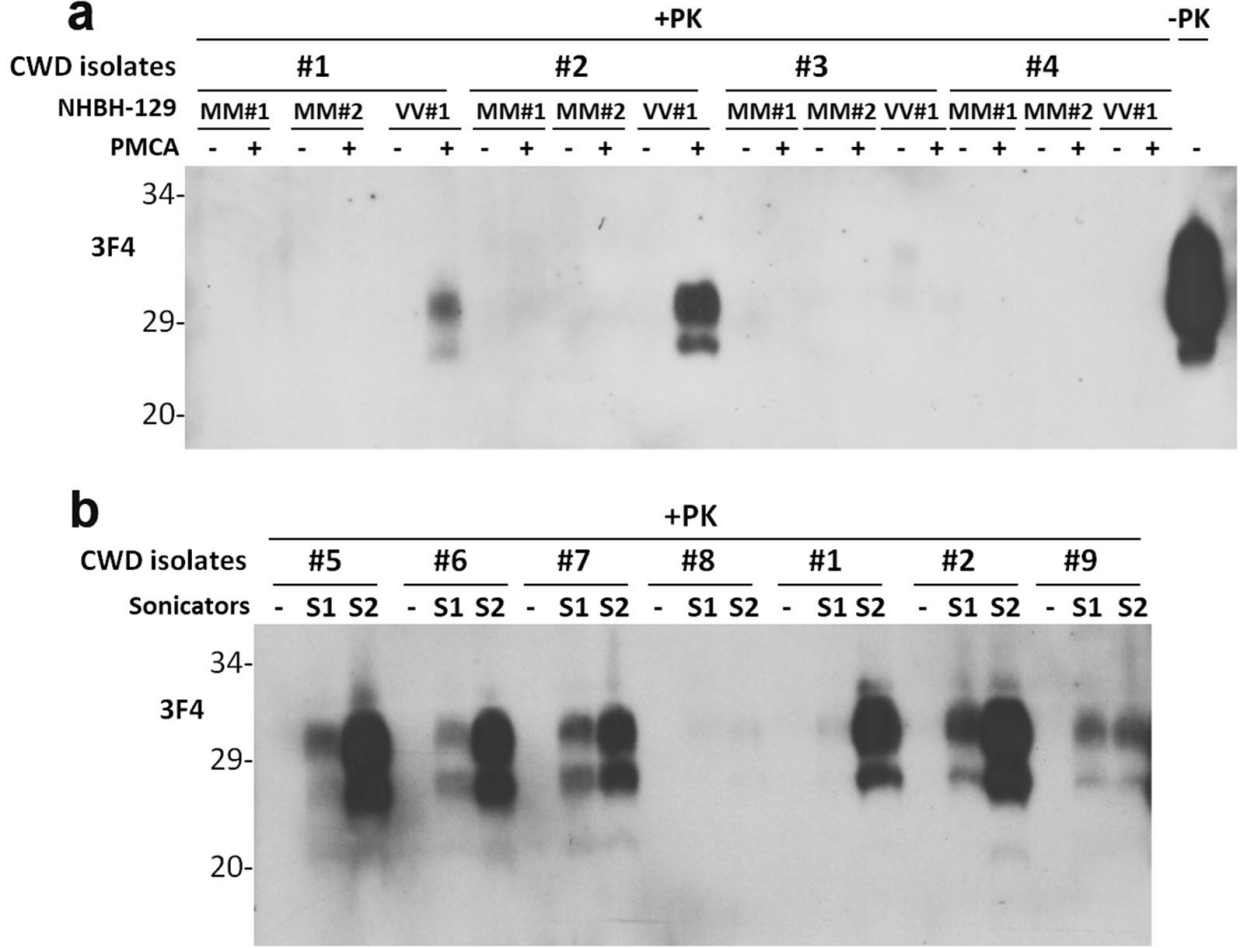
Western blotting of PK-resistant PrP^Sc^ in PMCA products. The PK-resistant PrP from PMCA products in the presence of non-CJD human brain homogenates containing PrP^C^ seeded with a trace amount of various CWD elk homogenates containing PrP^Sc^ was detected by western blotting probed with the monoclonal anti-PrP antibody 3F4. **a**, The PrP^C^ substrate used in the PMCA reaction was from human brain homogenates of non-CJD cadavers with either PrP^C^-129MM (MM#1 and MM#2 two individual cases) or PrP^C^-129VV (VV#1 case). The PrP^Sc^ seeds were from brain homogenates of three CWD elk cases (#1, #2 and #3) and a non-CWD elk (#4) was used as a negative control. **b,** The PrP^C^ substrate used in the PMCA reaction was from human brain homogenate of the non-CJD cadaver with PrP^C^-129VV (VV#1 case). The PrP^Sc^ seeds were from brain homogenates of six CWD elk cases (#5 through #8, #1, and #2) and a CWD deer case (#9). Two sonicators (S1 and S2) were used for PMCA as a comparison and duplication

To determine whether CWD prions from affected cervid animal brains can convert PrP^C^ from the normal human brain into PrP^Sc^, we conducted protein misfolding cyclic amplification (PMCA) assays. These PMCA assays used brain homogenates of 3 individual CWD elk (CWD prion isolates #1, #2, or #3) as the prion seeds and brain homogenates from three non-CJD subjects as the substrates, respectively. These non-CJD subjects included two cases homozygous for methionine (M) at polymorphic codon 129 of the PrP gene (PrP-129MM) termed MM#1 and MM#2, and a case homozygous for valine (V) at codon 129 (PrP-129VV) designated VV#1. After 1 round of PMCA, Western blotting showed PrP^res^ in 2 of 3 PMCA products seeded with CWD brain homogenates elk #1 and #2, but not elk #3 or the negative control elk #4, with the VV#1 substrate but not the MM#1 or MM#2 substrates (Fig. 1a). Since we used the anti-PrP monoclonal antibody 3F4 that is specific for human PrP but not cervid PrP as shown in Additional file 1: Fig. S1, we conclude that all detected PK-resistant PrP is exclusively human PrP^res^ instead of cervid PrP^Sc^, excluding the possibility that the detected PK-resistant PrP^res^ in the PMCA products could be the CWD PrP^Sc^ seeds themselves rather than newly-converted PrP^res^ from human PrP^C^.

To rule out the possibility that the detected PrP^res^ was de novo PrP^res^ generated from the PrP^C^ substrate itself by PMCA, we conducted serial PMCA (sPMCA) with normal brain homogenates in the absence of CWD PrP^Sc^ seeds (Additional file 1: Fig. S2). No PrP^res^ was detected in MM#1 and VV#1 after 12 rounds of sPMCA while it was detected in MM#2 after 6 or more rounds and in VV#2 after 9 or more rounds of sPMCA (Additional file 1: Fig. S2**a**, **c**, **d**). TgVV mouse brain homogenates showed no de novo PrP^res^ generation after 12 rounds while TgMM was observed to generate de novo PrP^res^ after 3 or more rounds of sPMCA (Additional file 1: Fig. S2**b**, **c**, **d**). In addition, normal hamster brain homogenates showed generation of de novo PrP^res^ after 6 or more rounds of sPMCA (Additional file 1: Fig. S1**c**) and no de novo PrP^res^ was found in TgDeer brain homogenates after 4 rounds of sPMCA (Additional file 1: Fig. S2**b**).

Using the same PMCA approach, we further examined the convertibility of human brain PrP^C^-129VV (VV#2) by PrP^Sc^ from 7 CWD cases that were composed of 6 CWD elks (#1, #2, #5-#8) and 1 CWD deer (#9). The PMCA assays were performed with two sonicators (S1 and S2) for comparison and duplication. We observed that PrP^Sc^ from all but one CWD elk isolate (#8) was able to convert human brain PrP^C^-129VV into PrP^res^ after one round of PMCA, as evidenced by the 3F4-detected PrP^res^ in the PMCA products (Fig. 1b). PrP^Sc^ from the CWD deer was also able to convert human brain PrP^C^ into PrP^res^ but the efficiency was lower compared to that of CWD elk (Fig. 1b). Moreover, the sPMCA-generated CWD-derived human PrP^Sc^ (Cd-HuPrP^res^) was consistently amplified until round 7 that showed a decrease in the amplification efficiency in both CWD isolates examined (Additional file 1: Fig. S3). Taken together, the results indicate that most of CWD prion isolates examined in this study were able to convert human PrP^C^-129 V but not human PrP^C^-129 M to generate Cd-HuPrP^res^ by PMCA in vitro, suggesting a possible polymorphism preference.

### Examination of infectivity of CWD-derived human PrP^Sc^ by animal transmission assay

**Fig. 2:**
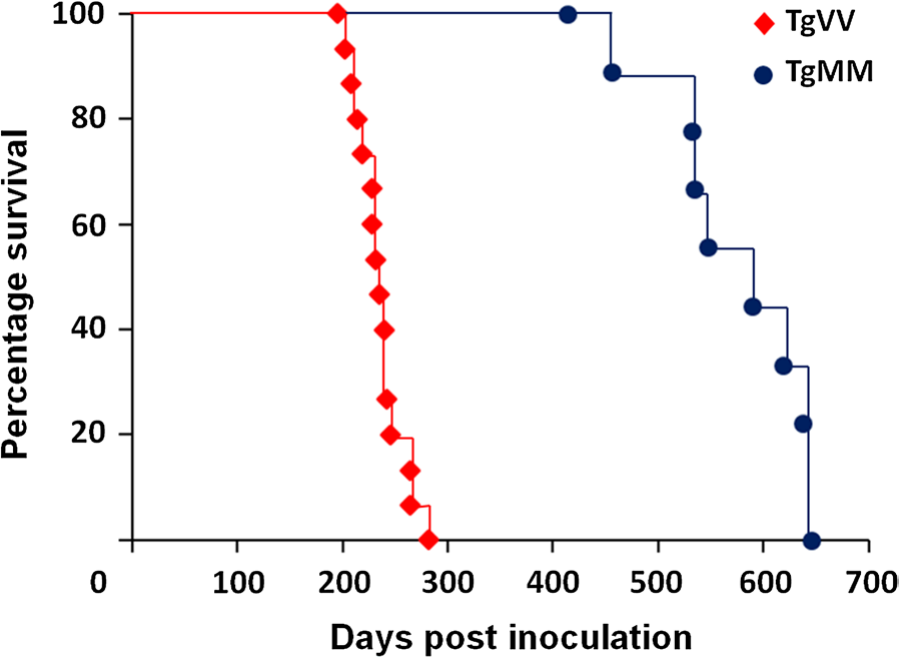
Kaplan–Meier survival plot for mice of inoculated TgMM or TgVV mice. 15 TgVV mice inoculated intracerebrally with diluted sPMCA product succumbed to the disease at an average of 233 ± 6 (SE) dpi (range,195 to 282 dpi) (red diamonds). 9 TgMM mice developed the disease at an average of 552 ± 27 (SE) dpi (range, 413 to 645 dpi) (blue circles), which showed a significantly longer incubation time than that of inoculated TgVV mice (*p* = 8.41465E-13 < 0.00001)

Next we intracerebrally inoculated the Cd-HuPrP^res^ (from the fourth round PMCA seeded the CWD elk #6 PrP^Sc^ seeds in the PrP^C^ substrate from the normal human brain homogenate of case VV#1) into humanized Tg mice. Two mouse lines were inoculated: one expressing wild-type human PrP-129VV (TgVV) or human PrP-129MM (TgMM, previously termed Tg40h) [20, 26]. All 15 TgVV mice inoculated intracerebrally with Cd-HuPrP^res^ developed disease at an average of 233 ± 6 (standard error, SE) days post inoculation (dpi) (range, 195 to 282 dpi) (Fig. 2). All 9 inoculated TgMM mice developed disease at an average of 552 ± 27 dpi (range, 413 to 645 dpi), a significantly longer incubation time than that of inoculated TgVV mice (*p* < 0.00001) (Fig. 2). This is not unexpected since the Cd-HuPrP^res^ was generated from the human VV#1 case and prior evidence supports that matching residue 129 between PrP^Sc^ and PrP^C^ facilitates prion propagation [19, 26].

**Fig. 3:**
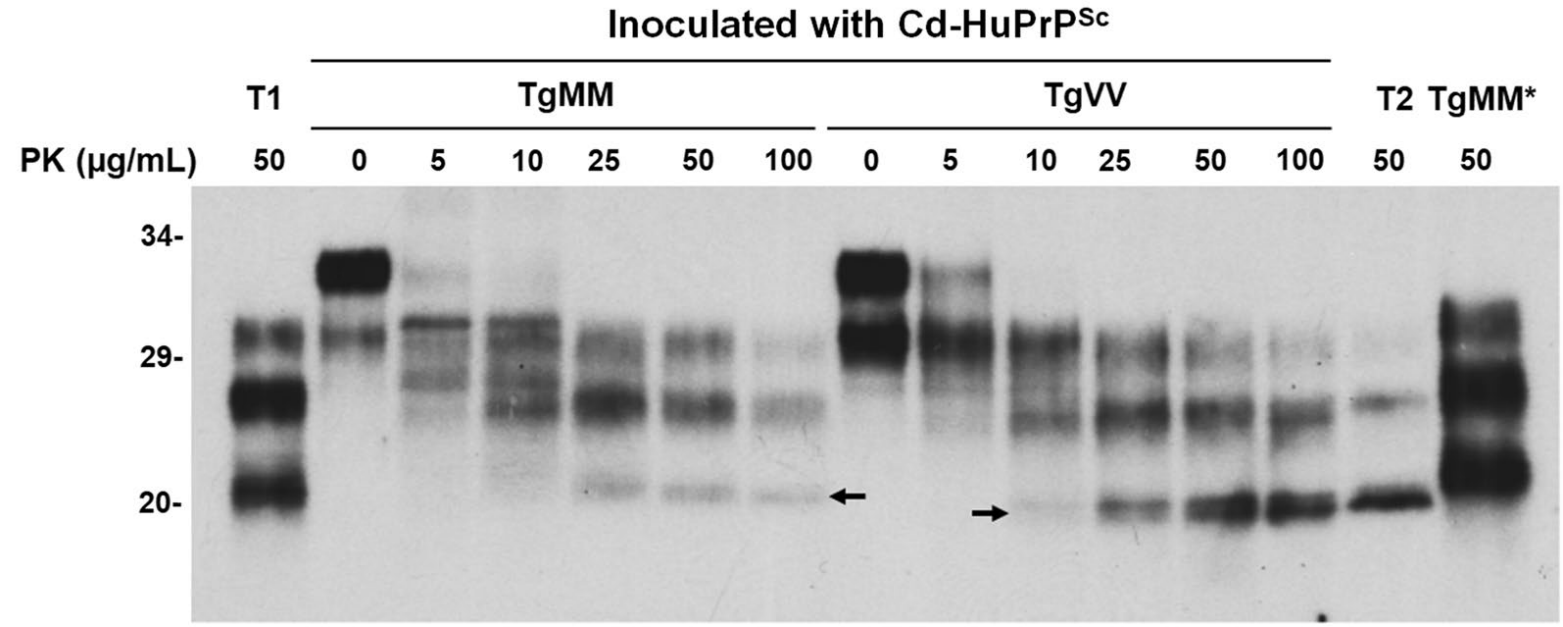
Comparison of PrP^Sc^ gel profile between humanized TgMM and TgVV mice inoculated with sPMCA-generated CWD-derived human prions (Cd-HuPrP^Sc^). Brain homogenates from TgMM and TgVV mice inoculated with Cd-HuPrP^Sc^ were treated with different amounts of PK ranging from 0 to 100 µg/mL prior to western blotting with the 3F4 antibody. Brain homogenates from patients with sCJDMM1 (T1) and sCJDMV2 (T2) were used as PrP^Sc^ type controls. The arrows indicate the gel mobility of the unglycosylated PK-resistant PrP^Sc^. TgMM*: PrP^Sc^ from TgMM mouse inoculated with sCJDMM1 used as a control

Brains of inoculated Tg mice were examined by western blotting and neurohistology for evidence of PrP^Sc^ deposition and spongiform degeneration. We compared the electrophoretic gel profiles of PrP^res^ from both Tg mouse lines inoculated with Cd-HuPrP^res^ after treatment of brain homogenates with a range of PK concentrations (0, 5, 25, 50, or 100 µg/mL). Interestingly, the gel mobility of PrP^res^ from the two lines of infected animals was different, in that TgVV displayed type 2-like PrP^res^ and TgMM exhibited type 1-like PrP^res^; however, the ratio of the diglycosylated to non-glycosylated PrP^res^ species was significantly higher in Cd-HuPrP^res^-infected TgMM and TgVV mice (Fig. 3, Additional file 1: Fig. S4), when compared to their sCJD counterparts PrP^res^ type 1 [9] [2.9 (n = 5) vs. 0.6 (n = 3), *p* < 0.001] and PrP^res^ type 2 [42] [2.1 (n = 7) vs 0.1 (n = 3), *p* < 0.001] (Additional file 1: Fig. S4).

**Fig. 4:**
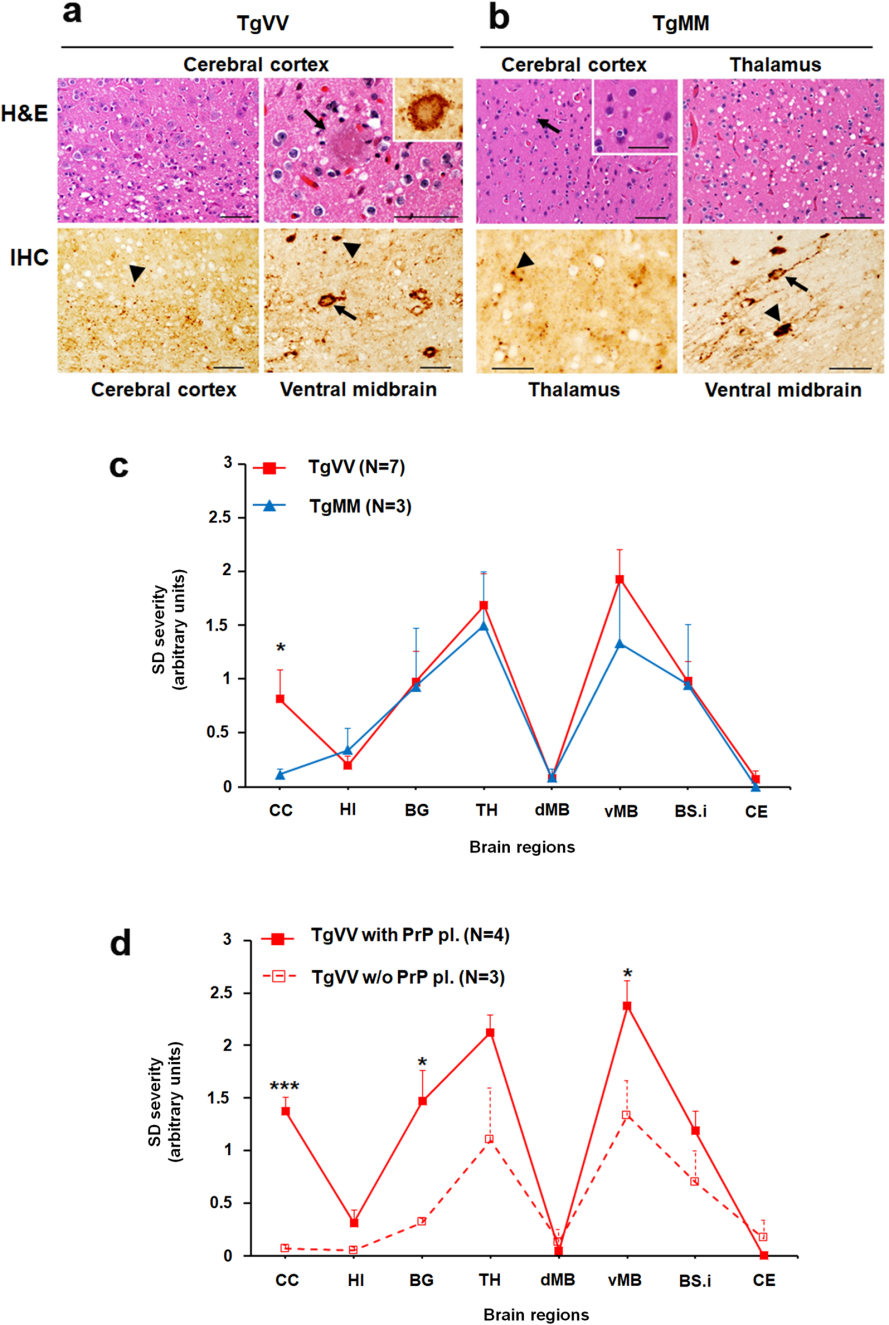
Hematoxylin and Eosin (H&E) staining and PrP immunohistochemistry (IHC). **a**, H&E (upper panels) & IHC (lower panels) staining of brain sections of TgVV mice inoculated with sPMCA-generated CWD-derived human PrP^Sc^ (Cd-HuPrP^Sc^). Upper left panel: Spongiform degeneration (SD) preferentially affecting the deep layers of the cerebral cortex (CC). Upper right panel: Cortical eosinophilic plaque surrounded by vacuoles; inset: PrP IHC of the plaque shown in the upper right panel; the rim of the plaque was more heavily stained than its core. Lower left panel: PrP granules (arrowhead) accumulating mainly in the deep cortical layers. Lower right panel: Granular PrP inside and around the perikarya and processes (arrow) and plaque-like PrP deposits (arrowhead). **b**, H&E (upper panels) & IHC (lower panels) staining of brain sections of TgMM mice inoculated with sPMCA-derived human CWD prions. Upper left panel: SD (arrow) affecting less severely CC than thalamus (Upper right panel); inset in Upper left panel: higher magnification of SD. Lower left panel: Granular PrP deposits affecting the thalamus; Lower right panel: Pattern of PrP deposition similar to those shown in TgVV mice (Lower left panel in a) affecting the ventral midbrain. Bar size: 50 µm; antibody: 3F4. **c**, Profiles of brain distribution and severity of SD in the two Tg mouse lines challenged with Cd-HuPrP^res^ were virtually identical, except for more severe lesions in the CC of TgVV mice. **d**, More severe lesions correlated with the presence of PrP plaques in TgVV mice; *P < 0.05–0.03, ***P < 0.006. HI: hippocampus, BG: basal ganglia, TH: thalamus, dMB and vMB: dorsal (d) and ventral (v) midbrain (MB), BS.i.: brainstem, inferior, CE: cerebellum. PrP pl.: PrP plaques; w/o: without. Each point of the lesion profile was expressed as mean ± standard error of the mean. Statistical significance was determined by a two-tailed Student’s *t*-test

The major histopathological differences between the two lines of inoculated mice were the significantly more severe cortical spongiform degeneration (SD) and the presence of plaque deposits in TgVV mice, compared with TgMM mice (Fig. 4a, b). The plaques were visualized on hematoxylin–eosin stained slides and confirmed to be composed of PrP by IHC staining with anti-PrP antibody 3F4 (Fig. 4a). Notably, occasional plaques were surrounded by vacuoles (Fig. 4a and Additional file 1: Fig. S5), resembling the florid plaques of CWD-affected cervids [24]. IHC staining also revealed granular PrP^Sc^ deposits that co-distributed with SD. Unlike TgVV mice, TgMM mice were free of plaque-like deposits in the cerebral cortex (CC). Lesion profiling showed similar SD distributions in the two groups of mice, except for the sparse presence of vacuoles in the cerebral cortex and overall less severe SD in the TgMM mice (Fig. 4c). Among the diseased TgVV mice, more severe lesions were found in animals bearing plaques than in those that were free of plaques (Fig. 4d). Moreover, in comparison with mice expressing matched PrP codon 129 genotypes but inoculated with sCJD prions [9, 42], the Cd-HuPrP^res^-infected TgVV and TgMM mice showed distinctive histopathological features. These features included (1) the presence of plaques reminiscent of CWD-florid plaques in some Cd-HuPrP^res^-infected TgVV mice; (2) seemingly more severe cortical spongiosis in Cd-HuPrP^res^-infected TgVV mice than in sCJDVV2-infected TgVV mice [9]; (3) lack of PrP plaques in our Cd-HuPrP^res^-infected TgMM mice but not in sCJDVV2-infected TgMM mice [9]; and (4) quite distinct lesion profiles between Cd-HuPrP^res^-infected TgMM mice and sCJDMM1-infected TgMM mice [42]. These data demonstrate that Cd-HuPrP^res^ is infectious and capable of inducing bona fide prion diseases in mice. We will term it as “Cd-HuPrP^Sc^” from here on.

It is conceivable that the sPMCA-generated infectivity in the above experiments could have resulted from de novo generation of PK-sensitive prions produced by sPMCA from the normal human brain homogenate VV#1 substrate rather than from CWD-seeded human PrP conversion. To rule out this possibility, we determined whether sPMCA induced an increase in the level of the insoluble PrP compared to that of normal brain homogenate substrate without PMCA treatment. We found that 4 rounds of sPMCA in the absence of CWD seeds actually led to a slight decrease of insoluble PrP in the P2 fraction and no detectable PK-resistant PrP^Sc^ (Additional file 1: Fig. S6), strongly suggesting that the observed infectivity of CWD-seeded sPMCA products in humanized mice was highly unlikely to be due to de novo generation of PK-sensitive PrP^Sc^.

## Discussion

Our current study has made the following findings. First, PMCA reveals that elk or deer CWD prions are able to overcome species barrier directly converting human brain PrP^C^ carrying 129 V but not 129 M polymorphism into PrP^Sc^ in vitro. Second, the PMCA-induced CWD-derived human PrP^Sc^ conformer (Cd-HuPrP^Sc^) is infectious, as it induced clinical prion disease in two lines of humanized Tg mice expressing human PrP with either 129 V or 129 M. Finally, PrP^Sc^ in brains of the diseased humanized Tg mice exhibits the electrophoretic mobility similar to those of sCJDMM1 or sCJDVV2 subtype, but the PrP^Sc^ glycoform ratios and neuropathological patterns are different. Histopathologically, the Cd-HuPrP^Sc^-infected TgVV and TgMM mice showed some distinctive features when compared to the Tg mice expressing the matched 129 genotypes but inoculated with sCJDMM1 or sCJDVV2 prions [9, 42]. Our findings raise several important issues and implications as to the potential transmissibility of CWD to humans involving effects of seeds and substrates on transmissibility as well as clinical phenotypes of human CWD and traits of CWD-derived human prion strains.

PMCA has been used not only for detection of small amounts of PrP^Sc^ in peripheral tissues and various body fluids of suspected prion-infected individuals but also for generation of new PrP^Sc^ [11, 12, 15, 39, 40], Moreover, it has been applied to explore the potential for cross-species conversion of PrP^C^ to PrP^res^ as a way to assess the susceptibility of human PrP^C^ to animal prion strains including CWD prions [2, 10, 17, 22]. Using PMCA, an earlier study revealed that wild-type human PrP^C^-129MM from humanized Tg mice was converted into human PrP^res^, but only after the CWD prion isolate had been stabilized by successive passages in cervid brain homogenates [4]. Moreover, the newly-generated PrP^res^ had unique biochemical properties with a glycoform pattern similar to CWD seeds but slower migration compared to the seeds. Subsequently, Barria et al. observed that elk CWD prions could convert PrP^C^ from human brain, humanized Tg mouse brain and cultured mammalian cells [2]. They also observed that the efficiency of in vitro cell-free human PrP conversion by cervid CWD prions was influenced by the PrP polymorphism at residue 129 in humans and its equivalent (residue 132) in cervids [3]. However, another study observed that mule deer PrP^Sc^ was unable to convert human PrP^C^ from humanized Tg mouse brain homogenates [23].

PMCA under properly optimized conditions has been believed to faithfully amplify prions from different species in vitro [29]. The parental prion seed strain traits have been observed to remain in the PMCA amplified prion products [11]. PMCA has become a useful approach to address fundamental questions about prion biology in vitro such as the molecular basis of the seeding activity, some aspects of species barrier and prion strain adaptation, and the role of cofactors on prion replication. It has been also used to assess the zoonotic potential of animal prion diseases such as BSE, scrapie, CWD, and atypical prion diseases. For instance, studies have confirmed that PMCA exhibits species specificity that faithfully reflects the same transmission barrier observed in animals in vivo [1, 10, 16, 28]. When BSE and scrapie were examined for their capability to convert human PrP with the three major polymorphic variants (*PRNP* codon 129 MM, MV and VV) expressed in the humanized transgenic mouse brain [5, 17], cattle BSE prions was able to trigger the efficient conversion of human PrP with a preference similar to that of human vCJD (MM > MV > VV) while scrapie failed to convert the human substrates [17]. These results suggest that PMCA can faithfully replicate aspects of cross-species transmission potential and might provide useful additional information concerning the molecular barrier to zoonotic transmission. Therefore, although in vitro seeded PrP^Sc^ amplification by PMCA may not mimic all aspects of in vivo conversion of brain PrP^C^ into PrP^Sc^, our finding of the CWD-induced conversion of human brain PrP^C^ into PrP^Sc^ suggests the potential of transmission of CWD to humans. It may also provide a model to dissect the mechanisms or factors that may be involved in the potential conversion of human PrP^C^ into PrP^Sc^ by the CWD prions.

The new Cd-HuPrP^Sc^ generated by seeding CWD isolates in human PrP^C^-129VV exhibits a PrP electrophoretic profile similar to that of the CWD PrP^Sc^ seeds that are observed to exhibit a more pronounced di-glycosylated PrP glycoform but an underrepresented unglycosylated PrP migrating at ~ 21 kDa [43]. Interestingly, it remains as the PrP^Sc^ type 1-like form when it is inoculated into the humanized TgMM mice, whereas it switches into the PrP^Sc^ type 2-like form when it is inoculated into the humanized TgVV mice. This phenomenon is consistent with the previous findings that PrP^Sc^ type 2 was only detected in Tg mice expressing human PrP-129 V inoculated with PrP^Sc^ sCJDMV2 or sCJDVV2 while only PrP^Sc^ type 1 was detected in mice homozygous or heterozygous for Met at residue 129 regardless of inoculated PrP^Sc^ types [7]. Moreover, PrP^C^-129MM substrates from two individual non-CJD human brain samples were not able to be converted by CWD prions in PMCA reactions, which contrasts with the observations of Barria et al. [2]. One possible explanation is that there are other co-factors or inhibitors affecting the PrP convertibility in the brain tissues of some subjects. This is consistent with the observations that although millions of people are believed to have been exposed to BSE-contaminated products in the European countries, especially in the UK, there were only about 229 vCJD cases reported worldwide as of 2015 [25]. It will be interesting to determine the convertibility of brain PrP^C^ from more normal human subjects including all three PrP genotypes (129MM, 129VV and 129MV).

The conversion efficiency varied between CWD prion isolates. Of the eight CWD isolates tested in this study, the seeding efficiency of #5 and #2 were the highest, followed by #6 and #1, then #7, #9, and the lowest were #8 and #3. Notably, match or mismatch between residue 129 of the human PrP substrate and residue 132 of the elk PrP^Sc^ seeds has been observed to affect the conversion efficiency in PMCA [3]. It will be important to further characterize the effect of different polymorphisms in CWD prion seeds and human PrP^C^ substrates on the conversion efficiency. Except for the difference in conversion efficiency, no other significant differences were detected in the electrophoretic gel profile of Cd-HuPrP^Sc^ derived from different CWD isolates.

Although our study clearly shows that CWD prions are capable of converting human PrP^C^ into infectious PrP^Sc^ in vitro, the real-world implications are less clear due to several factors. First, the CWD-induced conversion of human PrP^C^ into PrP^Sc^ was facilitated by repeated rounds of sonication in the PMCA procedure, which is unnatural. Second, in vivo prion clearing and selection mechanisms do not exist in the PMCA reactions, potentially making it easier for the newly converted PrP^Sc^ (including those that may be normally selected against in vivo) to persist and amplify in vitro. This could lead to the generation of PrP^Sc^ that is different from PrP^Sc^ generated in humanized mice after direct inoculation with brain homogenate from a CWD infected deer. Third, the PMCA substrates were derived from brain homogenates of only 4 non-CJD cases with PrP-129MM or -129VV. Finally, only a few CWD isolates from a limited number of cervid *PRNP* genotypes were examined. This makes it difficult to draw conclusions on the impact that other CWD strains and *PRNP* polymorphisms may have on the ability to convert human PrP^C^. More research will be needed to address these limitations.

In summary, our study demonstrates that CWD prions are able to cross the species barrier to convert human brain PrP^C^ into infectious PrP^Sc^ in vitro. Although the Cd-HuPrP^Sc^ largely retains the glycoform pattern of the CWD prion seeds, they produced type 1 PrP^res^ in the TgMM mice and type 2 PrP^res^ in the TgVV mice, but with distinct PrP^Sc^ glycoform ratios and histopathological features. We believe that our findings establish a new venue to study the likely molecular and neuropathological features of potential acquired human CWD cases, which may provide critical clues for identification of the first human cases of CWD infection should they occur.

## Supplementary Information

 **Additional File 1: Fig. S1.** Western blot analysis of PMCA-generated CWD-derived human PrP^Sc^ (Cd-HuPrP^Sc^) probed with 6D11 and 3F4. Representative Western blotting of the PK-treated (100 µg/mL) products of 4 rounds of sPMCA that was conducted with (+) or without (−) CWD isolate #7 seeds in the normal human brain homogenates with PrP-129VV (VV#1) probing with 6D11 (**a**) and 3F4 (**b**), respectively. The first lane is PrP^C^ from the normal human brain homogenate used as the substrate in sPMCA while the last lane is the PrP^Sc^ from the brain of CWD infected elk used as the seed. Both were directly loaded into the gel as the controls. **Fig. S2.** Western blot analysis of de novo generation of PK-resistant PrP^Sc^ by serial PMCA in the presence of PrP^C^ substrate from different species. Representative western blotting of PK-treated (100 µg/mL) products of sPMCA that was conducted with normal brain homogenates from different species in the absence of PrP^Sc^ seeds. The normal brain homogenates were from non-CJD cadaver brain tissues with PrP polymorphism methionine (M)/M (n=2, MM#1 and MM#2) or valine (V)/V (n=2, VV#1 and VV#2) at codon 129 of human PrP gene (PRNP), hamster, cervidized Tg mice (TgDeer) and humanized Tg mice (TgMM and TgVV). **a** and **b**, 1–4 rounds of sPMCA products with brain homogenates of MM#1, MM#2, VV#1, VV#2, hamster, TgMM, TgVV and TgDeer as the substrate, respectively. **c**, 5–8 rounds of sPMCA with brain homogenates of MM#1, MM#2, VV#1, VV#2, hamster, TgMM, and TgVV as the substrate, respectively. **d**, 9–12 rounds of sPMCA with brain homogenates of TgVV, MM#1, MM#2, VV#1, and VV#2 as the substrate, respectively. Blots were probed with the 3F4 antibody. **Fig. S3.** Western blot analysis of PMCA-generated CWD-derived human PrP^Sc^ (Cd-HuPrP^Sc^). Representative Western blotting of the PK-treated (100 µg/mL) products of 2 to 7 rounds of sPMCA that was conducted by seeding CWD isolate #5 or #2 in the normal human brain homogenates with PrP-129VV, respectively. The blot was probed with the 3F4 antibody. **Fig. S4.** Western blot analysis of PrP^Sc^ from TgMM and TgVV mice inoculated with PMCA-generated CWD-derived human PrP^Sc^ (Cd-HuPrP^Sc^). Representative Western blotting of PrP^Sc^ from brain homogenates of TgMM (**a**, #6145-1, #6146-2, #6146-3, #6146-4, and #6147-4) and TgVV (**b**, #1, #2, #3, #5, #7, #109, and #110) treated with PK (50 µg/mL). Brain homogenates from patients with sCJDMM1 (T1) and sCJDMV2 (T2) were used as PrP^Sc^ type controls. Neg: human brain homogenate without PK-treatment while other samples were all treated with PK at 50 µg/mL. The blot was probed with the 3F4 antibody. **Fig. S5.** Plaque and plaque-like PrP formations in TgVV mice. **a** H.E. depicting a plaque (arrow) surrounded by vacuoles. **b** PrP IHC showing plaque-like PrP deposits (arrowhead). Bar size: 50 µm; antibody: 3F4. **Fig. S6.** Western blotting of PrP from S2 and P2 fractions after ultracentrifugation of sPMCA products. The products of 4 rounds of sPMCA with the CWD prion isolate (#7) seeds (lanes 5 and 6) or without seeds (lanes 3 and 4) in the presence of normal human brain homogenate (VV#1) were subjected to ultracentrifugation in 5% lysis buffer at 100,000 g for 1 h at 4 °C to obtain supernatant (S2) and pellet (P2) fractions prior to Western blotting with 3F4 before (**a**) and after (**b**) treatment with PK at 100 µg/mL. The normal human brain homogenate without seeds and sPMCA was used as a control in lanes 1 and 2.

## Data Availability

All materials used in this study will be made available subject to a material transfer agreement.
