# Supplementary material for: Generation of human chronic wasting disease in transgenic mice
Source: Acta Neuropathol Commun. 2021 Sep 26;9:158. doi: 10.1186/s40478-021-01262-y (PMC8474769; doi:10.1186/s40478-021-01262-y)

Generation of human chronic wasting disease in transgenic mice

Zerui Wang^1*^, Kefeng Qin^2*^, Manuel V. Camacho^1*^, Ignazio Cali^1*^, Jue Yuan^1^, Pingping Shen^1^, Justin Greenlee^3^, Qingzhong Kong^1,4,5,6§^, James A. Mastrianni^2§^, Wen-Quan Zou^1,4,5,6§^

^1^Department of Pathology, Case Western Reserve University School of Medicine, Cleveland, Ohio 44106, USA

^2^Department of Neurology and Center for Comprehensive Care and Research on Memory Disorders, the University of Chicago Pritzker School of Medicine, Chicago, Illinois, USA

^3^Virus and Prion Research Unit, National Animal Disease Center, USDA, Agricultural Research Service, 1920 Dayton Avenue, Ames, IA 50010, USA

^4^Department of Neurology, Case Western Reserve University School of Medicine, Cleveland, Ohio 44106, USA

^5^National Prion Disease Pathology Surveillance Center, Case Western Reserve University School of Medicine, Cleveland, Ohio 44106, USA

^6^National Center for Regenerative Medicine, Case Western Reserve University School of Medicine, Cleveland, Ohio 44106, USA

*Contributed equally to this work.

^§^Co-corresponding authors: WQZ: [wxz6@case.edu](mailto:wxz6@case.edu); JM: [jmast@neurology.bsd.uchicago.edu](mailto:jmast@neurology.bsd.uchicago.edu); or QK: [qxk2@case.edu](mailto:qxk2@case.edu)

**Supplementary information**

**Supplementary figure legend**

Fig. S1 Western blot analysis of PMCA-generated CWD-derived human PrP^Sc^ (Cd-HuPrP^Sc^) probed with 6D11 and 3F4. Representative Western blotting of the PK-treated (100 µg/mL) products of 4 rounds of sPMCA that was conducted with (+) or without (-) CWD isolate #7 seeds in the normal human brain homogenates with PrP-129VV (VV#1) probing with 6D11 (**a**) and 3F4 (**b**), respectively. The first lane is PrP^C^ from the normal human brain homogenate used as the substrate in sPMCA while the last lane is the PrP^Sc^ from the brain of CWD infected elk used as the seed. Both were directly loaded into the gel as the controls.

Fig. S2 Western blot analysis of *de novo* generation of PK-resistant PrP^Sc^ by serial PMCA in the presence of PrP^C^ substrate from different species. Representative western blotting of PK-treated (100 µg/mL) products of sPMCA that was conducted with normal brain homogenates from different species in the absence of PrP^Sc^ seeds. The normal brain homogenates were from non-CJD cadaver brain tissues with PrP polymorphism methionine (M)/M (n=2, MM#1 and MM#2) or valine (V)/V (n=2, VV#1 and VV#2) at codon 129 of human PrP gene (*PRNP*), hamster, cervidized Tg mice (TgDeer) and humanized Tg mice (TgMM and TgVV). **a** and **b**, 1-4 rounds of sPMCA products with brain homogenates of MM#1, MM#2, VV#1, VV#2, hamster, TgMM, TgVV and TgDeer as the substrate, respectively. **c**, 5-8 rounds of sPMCA with brain homogenates of MM#1, MM#2, VV#1, VV#2, hamster, TgMM, and TgVV as the substrate, respectively. **d**, 9-12 rounds of sPMCA with brain homogenates of TgVV, MM#1, MM#2, VV#1, and VV#2 as the substrate, respectively. Blots were probed with the 3F4antibody.

Fig. S3 Western blot analysis of PMCA-generated CWD-derived human PrP^Sc^ (Cd-HuPrP^Sc^). Representative Western blotting of the PK-treated (100 µg/mL) products of 2 to 7 rounds of sPMCA that was conducted by seeding CWD isolate #5 or #2 in the normal human brain homogenates with PrP-129VV, respectively. The blot was probed with the 3F4 antibody.

Fig. S4 Western blot analysis of PrP^Sc^ from TgMM and TgVV mice inoculated with PMCA-generated CWD-derived human PrP^Sc^ (Cd-HuPrP^Sc^). Representative Western blotting of PrP^Sc^ from brain homogenates of TgMM (**a,** #6145-1, #6146-2, #6146-3, #6146-4, and #6147-4) and TgVV (**b,** #1, #2, #3, #5, #7, #109, and #110) treated with PK (50 µg/mL). Brain homogenates from patients with sCJDMM1 (T1) and sCJDMV2 (T2) were used as PrP^Sc^ type controls. Neg: human brain homogenate without PK-treatment while other samples were all treated with PK at 50 µg/mL. The blot was probed with the 3F4 antibody.

Fig. S5 Plaque and plaque-like PrP formations in TgVV mice. **a**: H.E. depicting a plaque (arrow) surrounded by vacuoles. **b**: PrP IHC showing plaque-like PrP deposits (arrowhead). Bar size: 50 µm; antibody: 3F4.

Fig. S6 Western blotting of PrP from S2 and P2 fractions after ultracentrifugation of sPMCA products. The products of 4 rounds of sPMCA with the CWD prion isolate (#7) seeds (lanes 5 and 6) or without seeds (lanes 3 and 4) in the presence of normal human brain homogenate (VV#1) were subjected to ultracentrifugation in 5% lysis buffer at 100,000 g for 1 hour at 4°C to obtain supernatant (S2) and pellet (P2) fractions prior to Western blotting with 3F4 before (**a**) and after (**b**) treatment with PK at 100 µg/mL. The normal human brain homogenate without seeds and sPMCA was used as a control in lanes 1 and 2.

**Supplementary figures**

Figure. S1


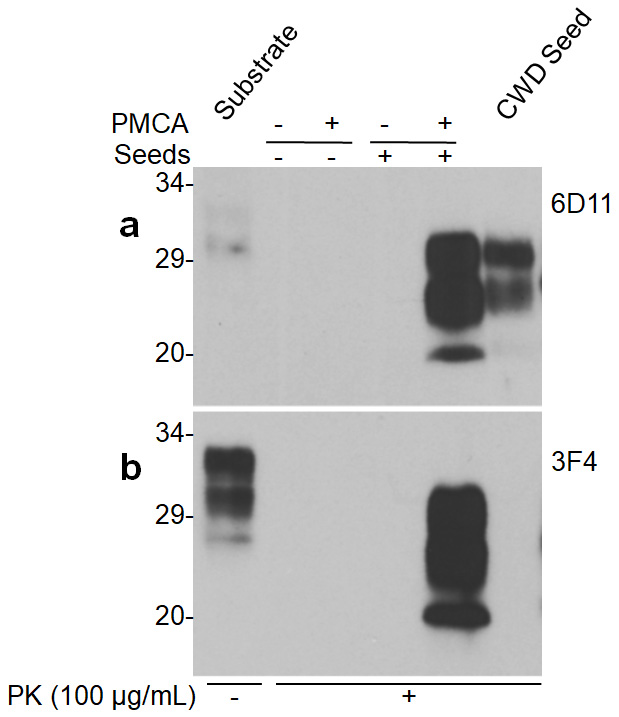


Figure. S2


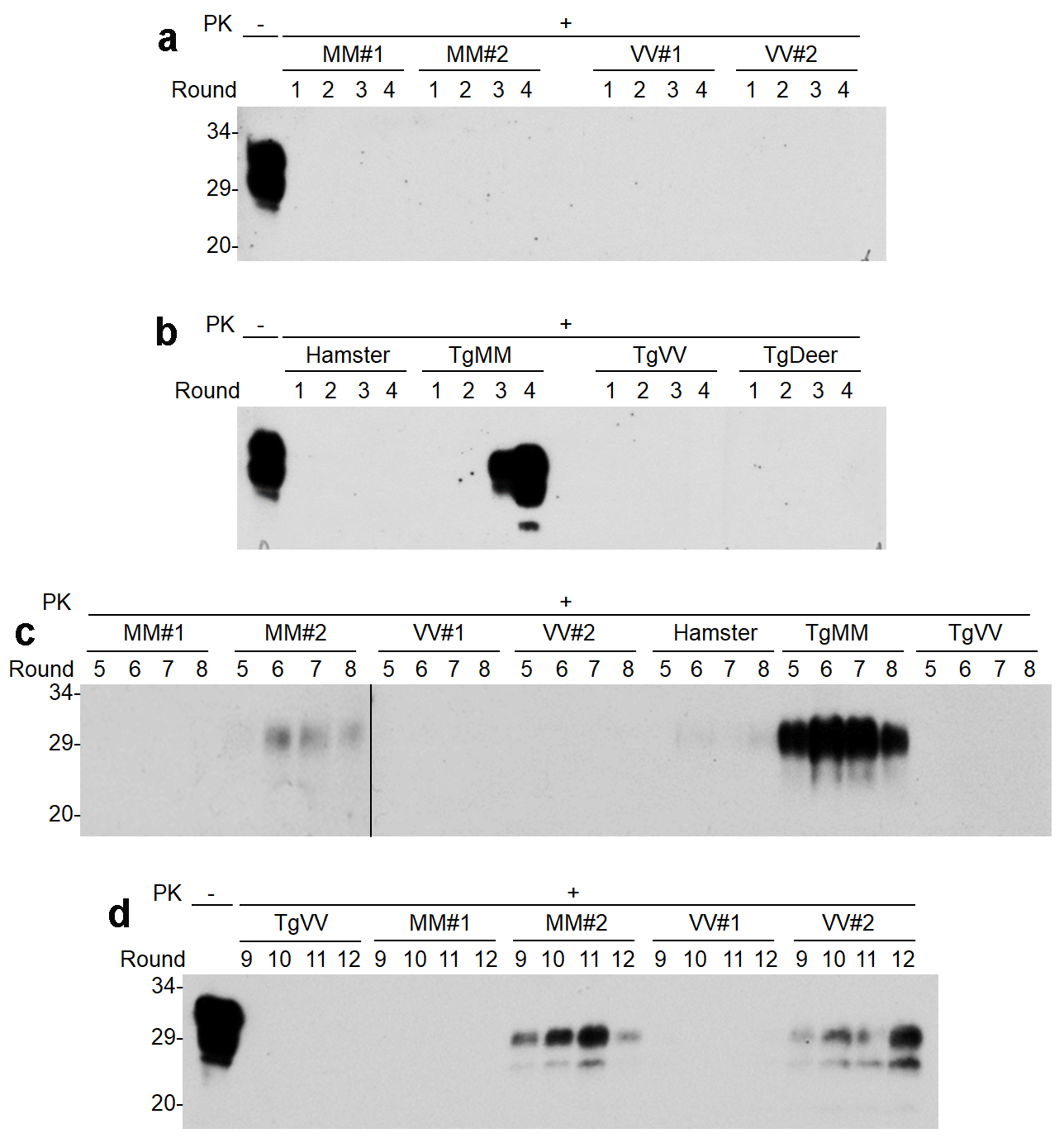


Figure. S3

**
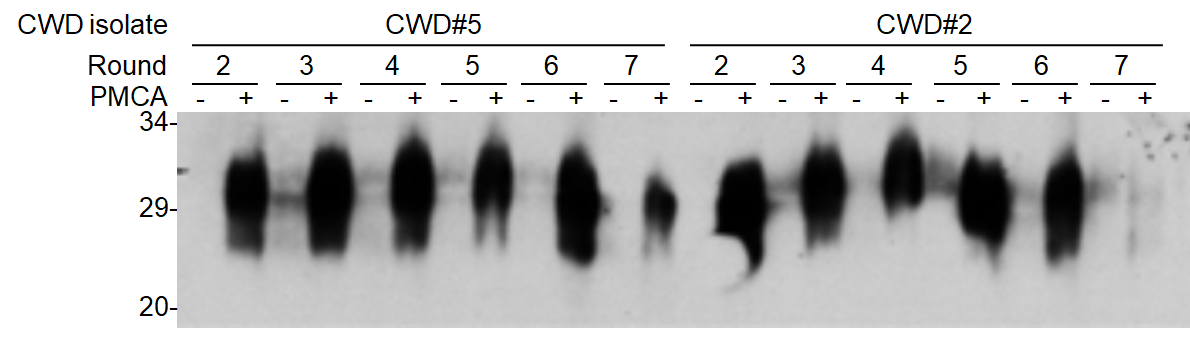
**

Figure. S4


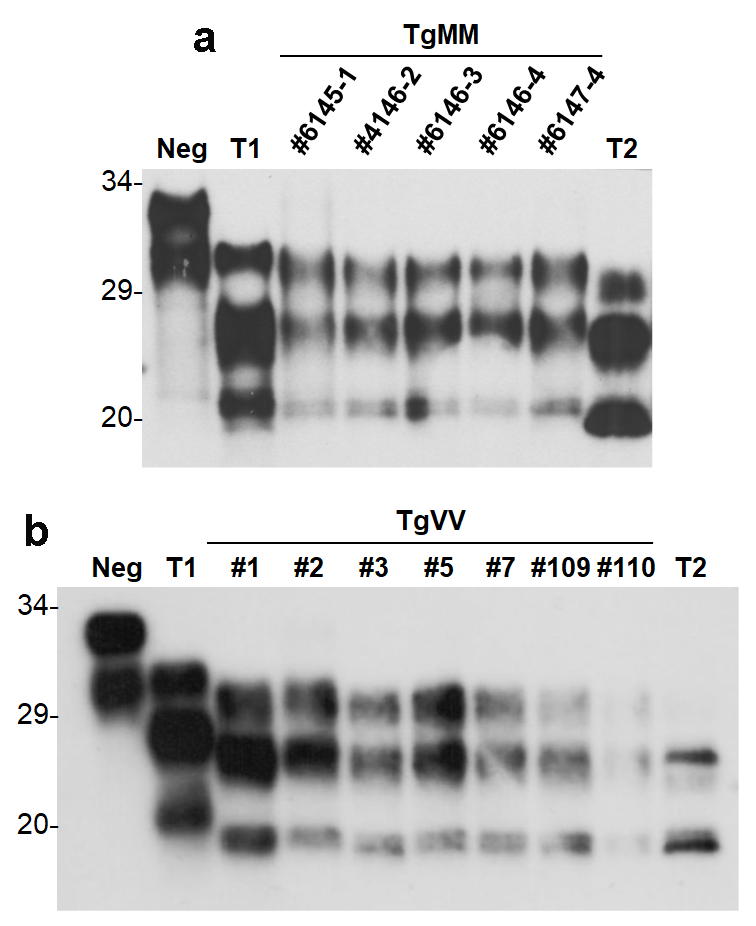


Figure. S5


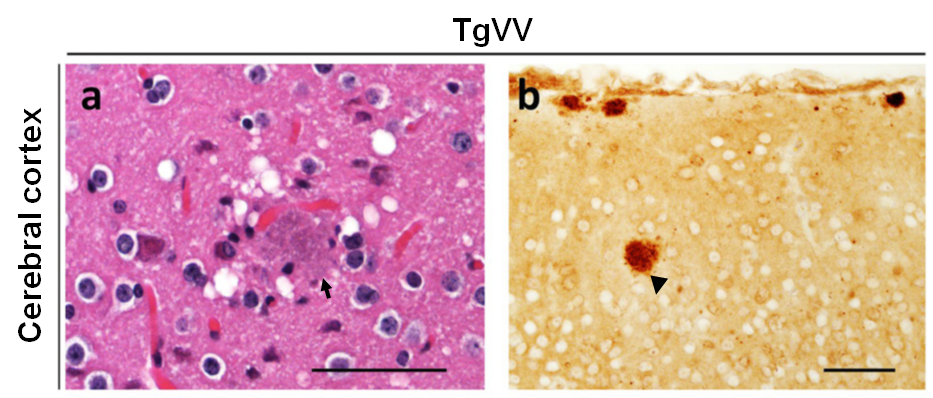


Figure. S6


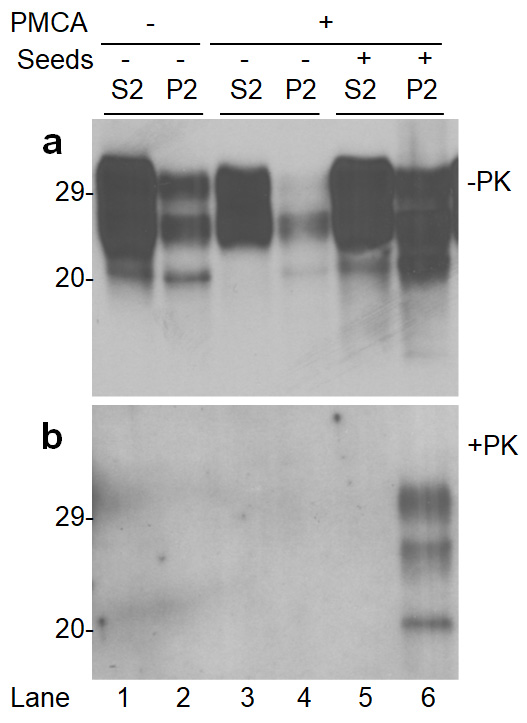

Supplement: Supplementary file 1 — Additional File 1: Fig. S1. Western blot analysis of PMCA-generated CWD-derived human PrPSc (Cd-HuPrPSc) probed with 6D11 and 3F4. Representative Western blotting of the PK-treated (100 µg/mL) products of 4 rounds of sPMCA that was conducted with (+) or without (−) CWD isolate #7 seeds in the normal human brain homogenates with PrP-129VV (VV#1) probing with 6D11 (a) and 3F4 (b), respectively. The first lane is PrPC from the normal human brain homogenate used as the substrate in sPMCA while the last lane is the PrPSc from the brain of CWD infected elk used as the seed. Both were directly loaded into the gel as the controls. Fig. S2. Western blot analysis of de novo generation of PK-resistant PrPSc by serial PMCA in the presence of PrPC substrate from different species. Representative western blotting of PK-treated (100 µg/mL) products of sPMCA that was conducted with normal brain homogenates from different species in the absence of PrPSc seeds. The normal brain homogenates were from non-CJD cadaver brain tissues with PrP polymorphism methionine (M)/M (n=2, MM#1 and MM#2) or valine (V)/V (n=2, VV#1 and VV#2) at codon 129 of human PrP gene (PRNP), hamster, cervidized Tg mice (TgDeer) and humanized Tg mice (TgMM and TgVV). a and b, 1–4 rounds of sPMCA products with brain homogenates of MM#1, MM#2, VV#1, VV#2, hamster, TgMM, TgVV and TgDeer as the substrate, respectively. c, 5–8 rounds of sPMCA with brain homogenates of MM#1, MM#2, VV#1, VV#2, hamster, TgMM, and TgVV as the substrate, respectively. d, 9–12 rounds of sPMCA with brain homogenates of TgVV, MM#1, MM#2, VV#1, and VV#2 as the substrate, respectively. Blots were probed with the 3F4 antibody. Fig. S3. Western blot analysis of PMCA-generated CWD-derived human PrPSc (Cd-HuPrPSc). Representative Western blotting of the PK-treated (100 µg/mL) products of 2 to 7 rounds of sPMCA that was conducted by seeding CWD isolate #5 or #2 in the normal human brain homogenates with PrP-129VV, respectively. The blot was probed wi [file 40478_2021_1262_MOESM1_ESM.docx]
